# Supplementary figures and images for: Adipose MSCs Suppress MCF7 and MDA-MB-231 Breast Cancer Metastasis and EMT Pathways Leading to Dormancy via Exosomal-miRNAs Following Co-Culture Interaction
Source: Pharmaceuticals (Basel). 2020 Dec 24;14(1):8. doi: 10.3390/ph14010008 (PMC7824212; doi:10.3390/ph14010008)

## Slide 1
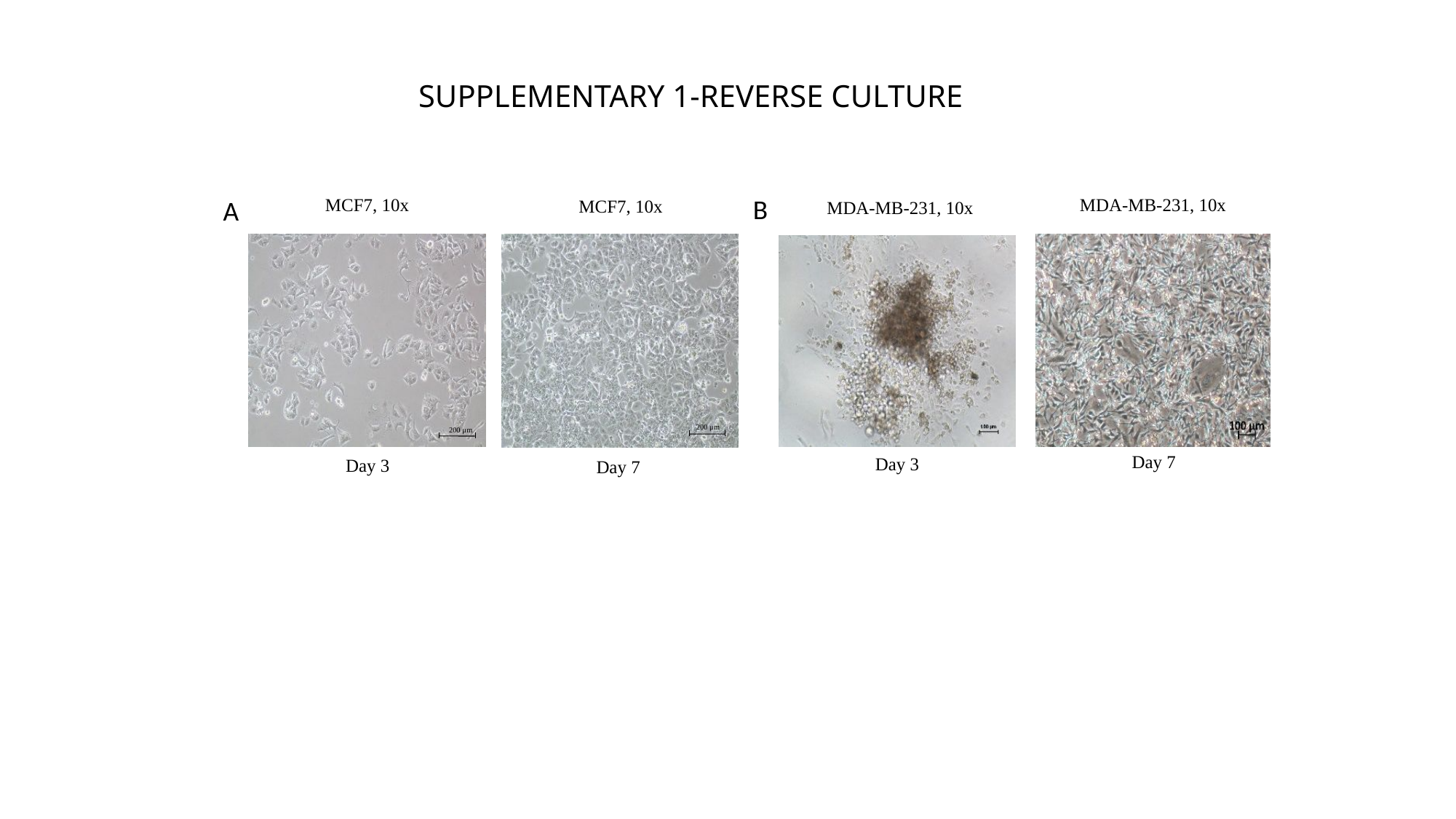

SUPPLEMENTARY 1-REVERSE CULTURE
MCF7, 10x
MDA-MB-231, 10x
B
MCF7, 10x
MDA-MB-231, 10x
200 μm
Day 7
Day 3
Day 3
Day 7
200 μm
A

Supplement: Supplementary file 1 [file pharmaceuticals-14-00008-s001.zip › Supplementary S1- Reverse Culture.pptx]
